# Impact of text contrast polarity on the retinal activity in myopes and emmetropes using modified pattern ERG

**DOI:** 10.1038/s41598-023-38192-9

**Published:** 2023-07-09

**Authors:** Sandra Wagner, Torsten Strasser

**Affiliations:** 1 Institute for Ophthalmic Research, University of Tuebingen, Elfriede-Aulhorn-Str. 7, 72076 Tuebingen, Germany; 2 University Eye Hospital Tuebingen, Elfriede-Aulhorn-Str. 7, 72076 Tuebingen, Germany

**Keywords:** Retina, Refractive errors, Electrophysiology

## Abstract

Environmental factors favoring myopia development are still being studied and there is accumulating evidence for a significant role of nearwork. Recently, reading standard black-on-white text was found to activate the retinal OFF pathway and induce choroidal thinning, which is associated with myopia onset. Contrarily, reading white-on-black text led to thicker choroids, being protective against myopia. Respective effects on retinal processing are yet unknown. Here, we exploratively assessed the impact of contrast polarity on the retinal activity and possible interactions with eccentricity and refractive error. We recorded pattern electroretinograms in myopic and emmetropic adults while presenting a dead leaves stimulus (DLS), overlaid by masks of different size in ring or circle shape, either filled with uniform gray or text of inverted or standard contrast. In myopes, retinal responses for DLS with standard and inverted contrast were larger when the perifovea was stimulated (6–12 deg), however, including the fovea resulted in smaller amplitudes for inverted contrast than in emmetropes. The retina of emmetropes was more sensitive to inverted contrast than to standard and gray within 12 deg, but most sensitive for gray in the perifovea. This demonstrates that the refractive error influences the sensitivity to text contrast polarity, with a special role of the peripheral retina, which is in line with previous studies about blur sensitivity. Defining whether the differences derive from retinal processing or anatomical features of a myopic eye requires further investigation. Our approach might be a first step to explain how nearwork promotes the eye’s elongation.

## Introduction

Myopia development is still being assessed in terms of genetic and environmental risk factors. As for the latter, numerous previous investigations provided evidence for an association between myopia onset, near vision, and reading^1–5^. This suggestion dates back to the seventeenth century, when Kepler already assumed that eyes exhibiting extensive amounts of nearwork might be at risk of developing myopia^6^. In recent decades, clinical trials supported this early hypothesis with the finding of a significant correlation between myopia prevalence and educational levels^7–9^, differing accommodation behavior in myopic compared to nonmyopic eyes^10–14^, specific anatomical and behavioral features of the myopic ciliary muscle^15–19^, and myopigenic dioptric and spatial frequency distribution in nearwork environment^20–23^. A recent investigation in young adults suggested that the reading material itself might influence the refractive development: Presenting text on a distant screen, it was found that eyes respond differently to standard contrast, with dark letters on bright background than to inverted contrast (bright text on dark): Standard contrast text predominantly activated the retinal OFF channels and induced a choroidal thinning, but overstimulation of retinal ON channels and choroidal thickening was measured with inverted contrast text^24^. The segregation of information processing into ON and OFF pathways serves the metabolically efficient signaling of luminance increments and decrements^25^. While photoreceptors and horizontal cells hyperpolarize with light, a sign reversal at the bipolar cell level allows the depolarizing ON type to respond to increments, the hyperpolarizing OFF cells to decrements of light^26^. The signal is transferred to amacrine and retinal ganglion cells, the latter being concentrically organized into ON-center/OFF-surround or vice versa^27^. Compared to ON cells, OFF cells are more numerous, have narrower receptive field sizes, a higher spatial resolution, lower sensitivity to luminance changes, and slower response kinetics^28–30^. Animal models revealed selective activation of these pathways to interfere with the refractive development^31–34^. Selective ON stimulation is further known to increase vitreal dopamine levels^35^. The choroid is also involved in the process of emmetropization: it thins with myopia onset, while a thickening might be protective from this development^36–39^. Reduced choroidal thickness was suggested to predict myopia in children^40^. Investigating young adults, Hoseini-Yazdi et al. reported that the accommodation-induced choroidal thinning increased with retinal OFF channel overstimulation. Moreover, the standard contrast text reading elicited a sustained choroidal thinning only in myopic, not in emmetropic eyes^41^. A psychophysical study with ON/OFF pattern detection provided further evidence for myopia being related to changes in the sensitivity to ON/OFF stimulation^42^. Up to the present, possible effects on retinal level resulting from selectively stimulating the two pathways have not yet been investigated in humans with emmetropia compared to myopia. The retina of myopic eyes provides lower amplitudes^43,44^ as well as increased latencies^45^ in electrophysiological tests. Multifocal electroretinography (mfERG) responses in children correlate with myopia progression^44^ and were suggested to serve as an early marker for myopia development^46^. The processing of contrast polarity information might therefore also differ in myopic compared to emmetropic retinae.

Previous electrophysiological studies on the ON/OFF pathways in humans mainly focused on glaucoma research^47^, applying full-field ERGs with sawtooth stimulation^48^ or steady-state visually evoked potentials to receive discrete ON and OFF responses^49,50^. The pattern ERG (pERG) was suggested as an alternative approach to assess the retinal ON/OFF system^47^. Using pERG, the effects of simulated optical blur on retinal responses, also considering different retinal eccentricities were recently investigated. Instead of the standard pERG protocol^51^, a novel dead leaves stimulus (DLS) was implemented. The results showed an increased sensitivity to blur in retinal areas between 6 and 12 deg, with no significant influence of refractive error^52^. Using real optical defocus, an analysis of mfERGs indicated that the blur signal is differentially decoded in the inner and outer retina, possibly enabling the sign of defocus discrimination^53^ necessary for adjusting the plane of focus during emmetropization^54^. It is yet unknown whether information processing of contrast polarity of text is, similar to defocus^55^, influencing eye growth. An assessment of retinal responses to stimuli of both polarities in myopic vs. nonmyopic eyes might, as a first step, reveal whether the retinal processing of the selective stimulation is affected by the refractive error. The following experimental approach allows, for the first time, to investigate the direct impact of contrast polarity on the retinal activity and the possible influence of eccentricity and refractive error.

## Results

Eight emmetropic (age 27.1 ± 8.2 years (s.d.); 4 male) and nine myopic healthy adults (age 30.7 ± 2.6 years (s.d.); 4 male) participated in this study. Monocular corrected Snellen visual acuity was 6/6 or better in each subject. Spherical equivalent refractive error of the right eye in the myopic group was −3.90 ± 3.52 D (s.d.) (range −12.63 D to −1.38 D), with astigmatism of −0.65 ± 0.38 D (s.d.) (range −1.25 D to −0.25 D) (sphere −3.72 ± 3.39 D (s.d.)). Myopic subjects were wearing their habitual correction during the ERG measurements (n = 3 soft contact lenses; n = 6 spectacle lens correction).

In the following, we concentrate on the findings where the 95% confidence intervals of the ERG waveforms of the mean differences (comparison of contrast polarity conditions) or the differences of the means (comparison of study groups) did not overlap, being also highlighted in the corresponding figures.

### Comparison between refractive groups

**Figure 1:**
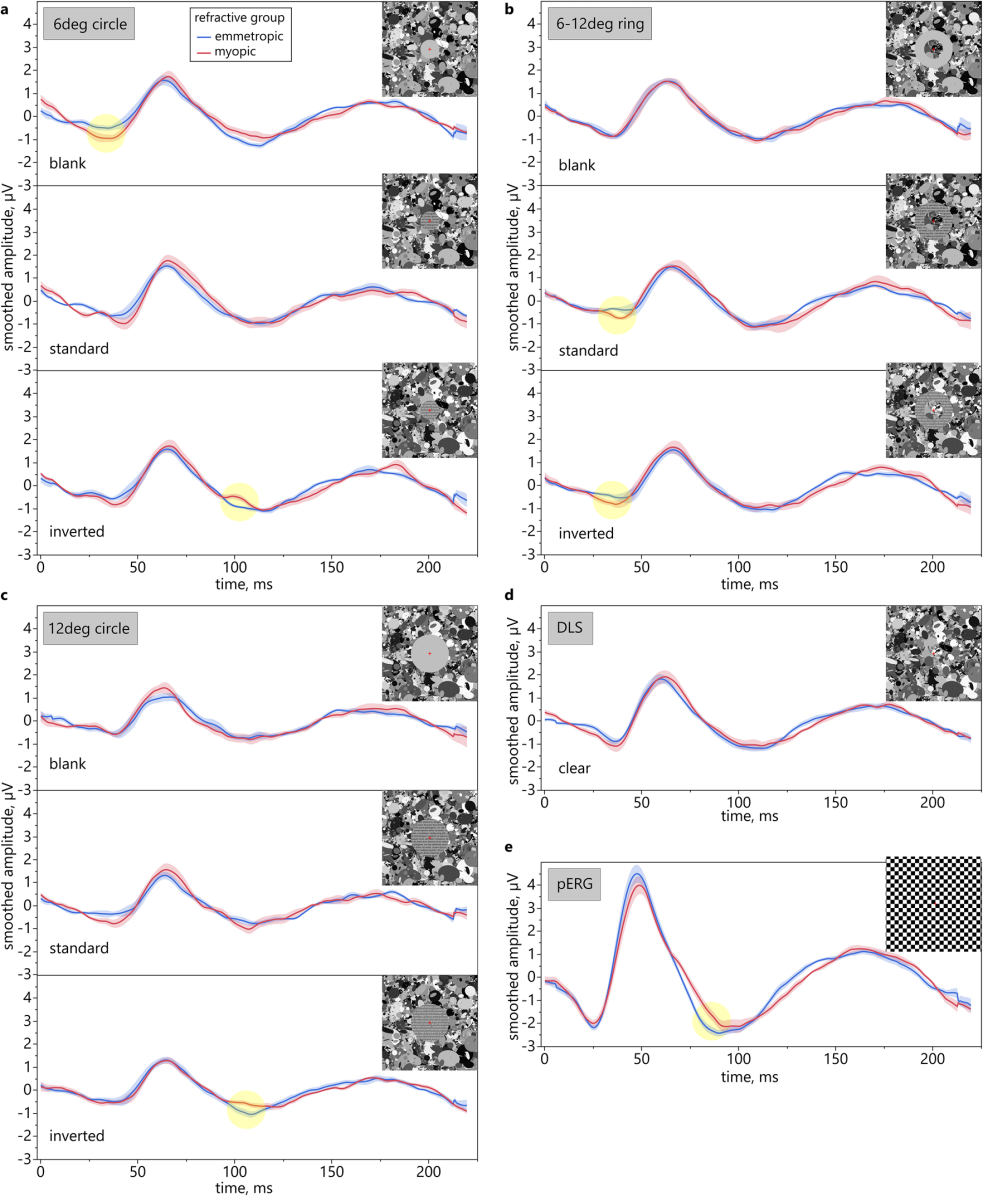
Comparison of ERG responses between refractive groups. ERG results of emmetropic (blue) and myopic subjects (red) for different stimulus sizes, shapes, and contrast polarities (smoothed averaged amplitude, shaded areas are SE, highlights mark substantial differences; see Supplementary Fig. 1).

A substantial reduction of the N95 amplitudes in the standard pERG was found in myopic compared to emmetropic eyes. In contrast, the clear DLS elicited similar retinal response levels in both refractive groups (Fig. 1d+e; Supplementary Fig. 1d+e). Differences between myopic and emmetropic ERGs were revealed when implementing the text components of different size and contrast in the DLS: Stimulation with both standard and inverted contrast text led to larger N1 amplitudes in myopes compared to emmetropes when presented perifoveally, within the area between 6–12 deg (Fig. 1b; Supplementary Fig. 1c). Substantially smaller N2 responses for the inverted contrast condition were measured in myopes in case of the text being presented with inclusion of the fovea for both stimulus sizes, 6 deg and 12 deg circles (Fig. 1a+c; Supplementary Fig. 1a+b). Regarding the blank condition, when masks were filled with uniform gray, the two groups differed considerably for foveal stimulation, with myopes providing increased N1 responses (Fig. 1a; Supplementary Fig. 1a).

### Comparison between contrast polarity conditions

**Figure 2:**
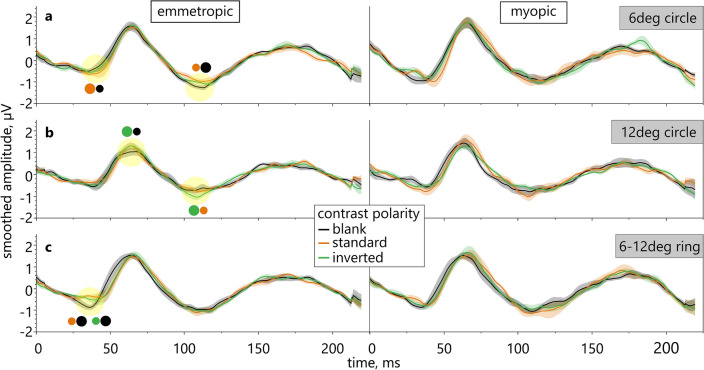
Comparison of ERG responses between contrast polarity conditions. ERG results for DLS overlaid with stimuli of different sizes, shapes, and content in emmetropic (left) and myopic eyes (right). Size of color-coded dots illustrates result of pairwise comparison between polarities (smoothed averaged amplitude, shaded areas are SE; highlights mark substantial differences; see Supplementary Fig. 2). Similar substantial differences were not revealed in myopic eyes.

A separate group analysis of the effects of contrast polarity and eccentricity showed that for the 6 deg circle and for the 6–12 deg ring stimulus size, standard and inverted contrast did not affect the retinal responses differently in either group (Fig. 2). However, in emmetropes, the stimulation with the 12 deg circle led to larger N2 responses for the inverted contrast condition than the standard contrast condition (Supplementary Fig. 2c). Furthermore, P1 responses were also substantially larger for the inverted contrast than the blank condition, i.e. when the circle was filled with uniform gray (Fig. 2b; Supplementary Fig. 2b). As for the 6 deg circle, compared to the blank condition, emmetropic eyes showed increased N1 amplitudes but decreased N2 amplitudes for the standard contrast condition (Supplementary Fig. 2a). If the fovea was excluded (6–12 deg ring), the emmetropic retina showed larger N1 responses for the blank condition than for both other conditions, namely standard and inverted contrast (Fig. 2c; Supplementary Fig. 2a+b). Thus, when changing the area of stimulation from 6 deg to 6–12 deg, the largest retinal reaction to the blank condition also changed in emmetropes from N2 to N1.

**Figure 3:**
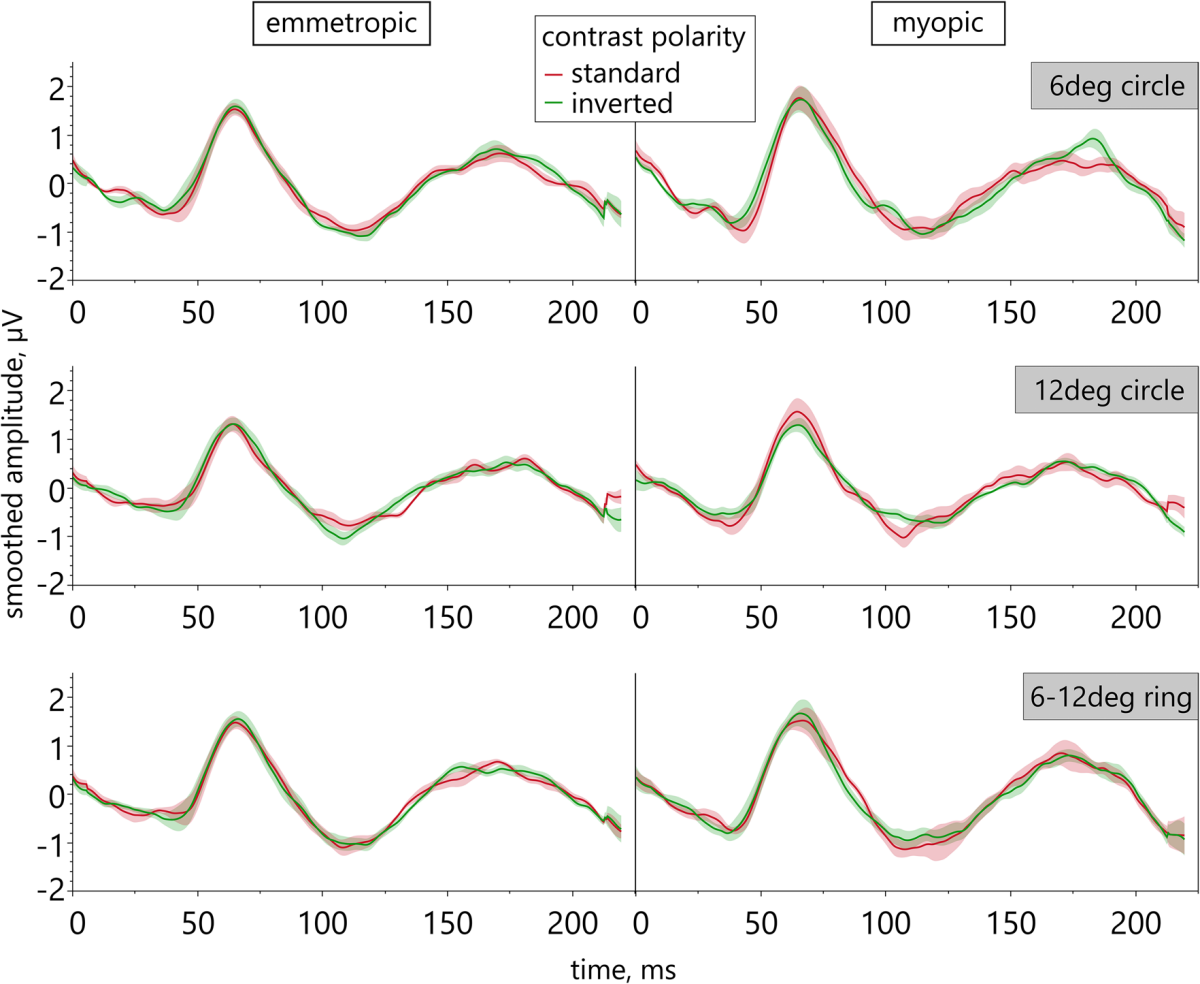
ERG responses for DLS with text of both contrast polarities. Effects of standard vs. inverted contrast text elements of different size in emmetropic (left) and myopic eyes (right; smoothed averaged amplitude, shaded areas are SE).

Narrowing the analysis to a comparison of the effects of text of standard vs. inverted contrast, separately in the two groups, reveals a difference in their retinal responses for a stimulation size of 12 deg (Fig. 3): The inverted contrast text induced an increased N2 amplitude in emmetropes, while myopic retinae reacted with an increased N2 amplitude to the standard contrast text stimulus. Only for this stimulus size, such a difference between the two refractive groups with respect to ON vs. OFF stimulation was present.

## Discussion

Reading and myopia development are undoubtedly associated, however, the reasons for close work eliciting the eye’s elongation have not yet been entirely fathomed. A predominant retinal OFF pathway stimulation together with choroidal thinning^24^, which is associated with myopia development, was previously measured after reading text with dark letters on bright background. Investigating the differences in retinal processing between eyes with myopia compared to nonmyopia during these stimulations might increase our knowledge on factors favoring myopia onset and improve current myopia management.

Using modified pERG with DLS and text elements, we analyzed the sensitivity to contrast polarity at different retinal eccentricities in myopic vs. emmetropic eyes. Results showed that contrast polarity of text affects ERGs differently depending on retinal eccentricity and refractive error. Compared to emmetropes, myopes had smaller retinal responses to inverted contrast for foveal and parafoveal, but not for perifoveal stimulation between 6–12 deg eccentricity. An interesting outcome regarding the emmetropic group was that at 12 deg, inverted contrast stimulation elicited considerably larger retinal responses than the blank and standard contrast conditions. Also, only in emmetropes, when changing the stimulation area from perifoveal to fovea only, the largest retinal activity to the blank condition switched from N1 to N2.

The results support the hypothesis that the retinal region between 6–12 deg holds differing characteristics from the foveal area: When using the ring mask, a reduced sensitivity to ON pathway stimulation was not present in myopic ERGs. Previously, the reaction to calculated blur was likewise found to be different within this area, revealing a higher sensitivity to blur, which was similar in emmetropes and myopes^52^. These findings confirm earlier mfERG recordings with ophthalmic lens defocus, showing that paracentral regions respond stronger to imposed defocus than central retinal regions^53^. Due to the lack of high spatial frequencies, the presence of blur could be compared to the blank condition in the present study, operating like a low-pass filter. We found similar responses to the blank stimulus in both study groups within the 6–12 deg area, however, not with respect to a stimulation size of 6 deg, i.e. the fovea only condition. Here, myopes showed a larger sensitivity than emmetropes to the stimulus of uniform gray. Conflicting outcomes were published in the past, with some authors reporting that myopes tolerate more blur than nonmyopes^56^, while others found a reduced blur sensitivity only for monocular viewing conditions^57^ or no dependence on refractive error^58^. Furthermore, in a recent psychophysical study, Xu et al. revealed an increased contrast sensitivity in myopes at 6 deg and 12 deg of the superior and inferior visual field, while there was no difference to nonmyopes foveally^59^. It is unclear whether the psychophysically measured sensitivity can be directly correlated to retinal activity in ERG and further assessments of this relationship are required. Since peripheral retinal image quality significantly influences refractive error development^60,61^, eccentricity is a factor that certainly needs to be considered in both psychophysical and electrophysiological visual tests.

A difference of the ERG responses between refractive groups for stimulation with inverted vs. standard contrast was only given when the stimulus was presented in the area until 12 deg, covering foveal and parafoveal regions (Fig. 3). While emmetropes showed increased retinal responses to inverted, myopes reacted stronger to standard contrast text. Increased sensitivity of myopic eyes to standard contrast text was likewise previously found in young adults using the choroidal thickness as indicating parameter: Only myopes exhibited sustained subfoveal choroidal thinning in response to a continuous 30-min OFF pathway stimulation after a 20-min recovery phase. This was suggested to be related to more potent OFF or less potent ON pathway-mediated signals in myopes^41^. However, a psychophysical assessment of contrast sensitivity for both contrast polarities provided contradicting results: Sensitivity was significantly reduced in myopic young adults when black letters were presented on gray background (negative contrast)^62^. In a later study assessing more subjects, the author reported increased contrast thresholds for negative than positive contrast in myopes and reverse outcomes in emmetropes^63^. In this investigation, the two conditions were not matched in their luminance, which might explain the deviance from our results and those of Hoseini-Yazdi et al.^41^.

A merely anatomical reason for the here presented finding of reduced ON sensitivity in myopes might be possible: The ON pathway is mainly active at lower temporal frequencies, while the OFF pathway predominates at higher frequencies^47,64^. Thorn et al. suggested that anatomical changes associated with stretching in the myopic retina might largely affect bipolar and ganglion cells of the Y-system, therefore leading to reduced sensitivity for moving gratings, high temporal frequencies, and low spatial frequencies. However, psychophysically measured temporal and spatial contrast sensitivity for static and moving gratings was not reduced in high myopic subjects up to −10 D. The authors concluded that even in high myopia, the normal integrity of the retina is preserved and only changes after occurrence of pathological events^65^. In the present study, with one exception, subjects were in the range of low to moderate myopia, rendering a sole anatomical explanation for the described differences unlikely.

In recent years, around 200 genetic loci for myopia have been identified in genome-wide association studies^66–69^, also revealing interaction effects with environmental factors, especially education and nearwork^70^. Considering contrast polarity as a phenotypic variance, the presented differences as for eccentricity and refractive error group might be associated with specific retinal areas being more genetically, others more environmentally driven, however, further genetic and psychophysical studies are required to investigate this idea.

To evaluate possible consequences of our findings about the sensitivity to contrast polarity at different retinal eccentricities, the reading process needs consideration: Reading requires a minimum visual field of 4 deg horizontally around the fixation point^71^, while the perceptual span can increase to 5 deg in reading direction^72^. In the current study, these areas were shown to be less sensitive to ON pathway stimulation in myopic eyes. This observation and previous post-nearwork choroidal thickness measurements^41^ lead to the hypothesis that reading black-on-white text might have an even higher potential to induce myopic progression in a myopic eye than to induce myopia onset in an emmetropic eye. Further supporting data arise from a recent investigation on the ciliary muscle, a structure most important for the accommodation process that was suggested to be involved in refractive error development^19,73^. We previously showed that, independent of the refractive error, the ciliary muscle undergoes a significant thinning after a prolonged reading period of standard black-on-white text^74^. Being positioned below the sclera and closely linked to the choroid, the muscle might also be influenced by the features of the image as it was found for choroidal thickness^24^. A preliminary study with six subjects revealed that after reading bright text on dark background, the ciliary muscle got thinner in myopes as shown before, while in emmetropes, muscles were rather thicker after the close work^75^. Contrast polarity of text might only be of importance prior to myopia onset, but without any impact after its development. Based on this hypothesis, an intervention in pre-myopic children, at most under the age of 7 years, before the typical beginning of school myopia, would be recommended, e.g. by introducing inverted contrast as new standard presentation mode already in kindergarten/ pre-school learning tools. The efficacy of these non-invasive interventions would certainly need to be evaluated in longitudinal pediatric trials.

Instead of a commonly used checkerboard limited to a single spatial frequency and fixed contrast, we used a DLS for recording the ERGs since it offers a broad range of spatial frequencies and a contrast ranging between 0 and 100%, which simulates the statistics of natural images^76^. Also, in contrast to the conventional assessment of ERGs using single markers, we applied point-wise t-testing for comparing the shape of the entire ERG responses over time. As illustrated in Supplementary Figs. 1 and 2, substantial differences between the tested stimuli conditions or study groups were assumed if the respective 95% confidence intervals of the waveforms did not overlap. Since it is an explorative study aiming at detecting the effects of both contrast polarity and eccentricity and the interaction with the refractive error, we did not correct for multiple testing. Also, to simplify, we assumed radially symmetric retinal sensitivity areas. Several approaches have recently been proposed to assess the retinal field size and properties of retinal ganglion cells (RGCs), including computational models^77^. Our simplification is supported by a mathematical formula correlating receptive field density of human RGCs with the position in the visual field, and showing that the density falls off linearly between 5 to 10 deg, both nasally and temporally^78^. A possible detriment of our methodology is that retinal responses derived from the pERG are a local response of the stimulated area and primarily originate from the ganglion cell activity^51^. In the mouse model, P1 was found to mainly represent the ON pathway and N2 the OFF pathway contributions^79^. In humans, P50 of transient pERG was suggested to originate from spiking and non-spiking activity of both pathways and N95 from their spiking activity only^80^, but an assignment of pERG components to specific retinal cell subtypes or structures cannot be easily realized. Thus, improved interpretation of the described observations would require the implementation of text elements in further ERG types (e.g. steady-state pERG, full-field ERG with On-Off^48^ and photopic negative response, long flash ERGs). Rather than selectively blocking the ON pathway using pharmaceuticals as in animal models^81^, this pathway’s contribution could be studied by including patients with complete congenital stationary night blindness (cCSNB), being characterized by an ON-bipolar cell dysfunction^82,83^. The described adjustments of the ERG protocol, together with a randomized stimulus presentation are planned in future studies with increased sample size. Although the presented differences between groups and conditions are small, they have the potential to provide the basis for further investigations that contribute to explaining why reading and nearwork promote myopia development, and how this causality can be prevented.

We introduced a novel pERG protocol to measure retinal responses during the selective stimulation of retinal ON and OFF pathways. Stimuli were composed of a DLS, containing a broad range of spatial frequencies and contrasts, and overlaying text elements of both contrast polarities in different size and shape. The recordings revealed that effects of text contrast polarity on retinal activity are influenced by eccentricity and refractive error. Retinal responses of myopic eyes were smaller than those of emmetropes during stimulation with inverted contrast in foveal and parafoveal regions, but not perifoveally around 6–12 deg. Further studies are planned to permit an improved distinction between retinal ON and OFF activity and to assess whether the presented effects arise from anatomical differences or different retinal processing in myopic eyes. A thorough electrophysiological investigation of the retinal ON/OFF pathway activation during nearwork might substantially improve our understanding of myopia development and current myopia management strategies. Given a causative relationship between contrast polarity and myopigenesis and regarding the abundant daily use of digital devices throughout a child’s school day and leisure time^84^, using reading material in the inverted contrast form might be a simple implementable method to support myopia management treatment in the future.

## Methods

### Stimuli

**Figure 4:**
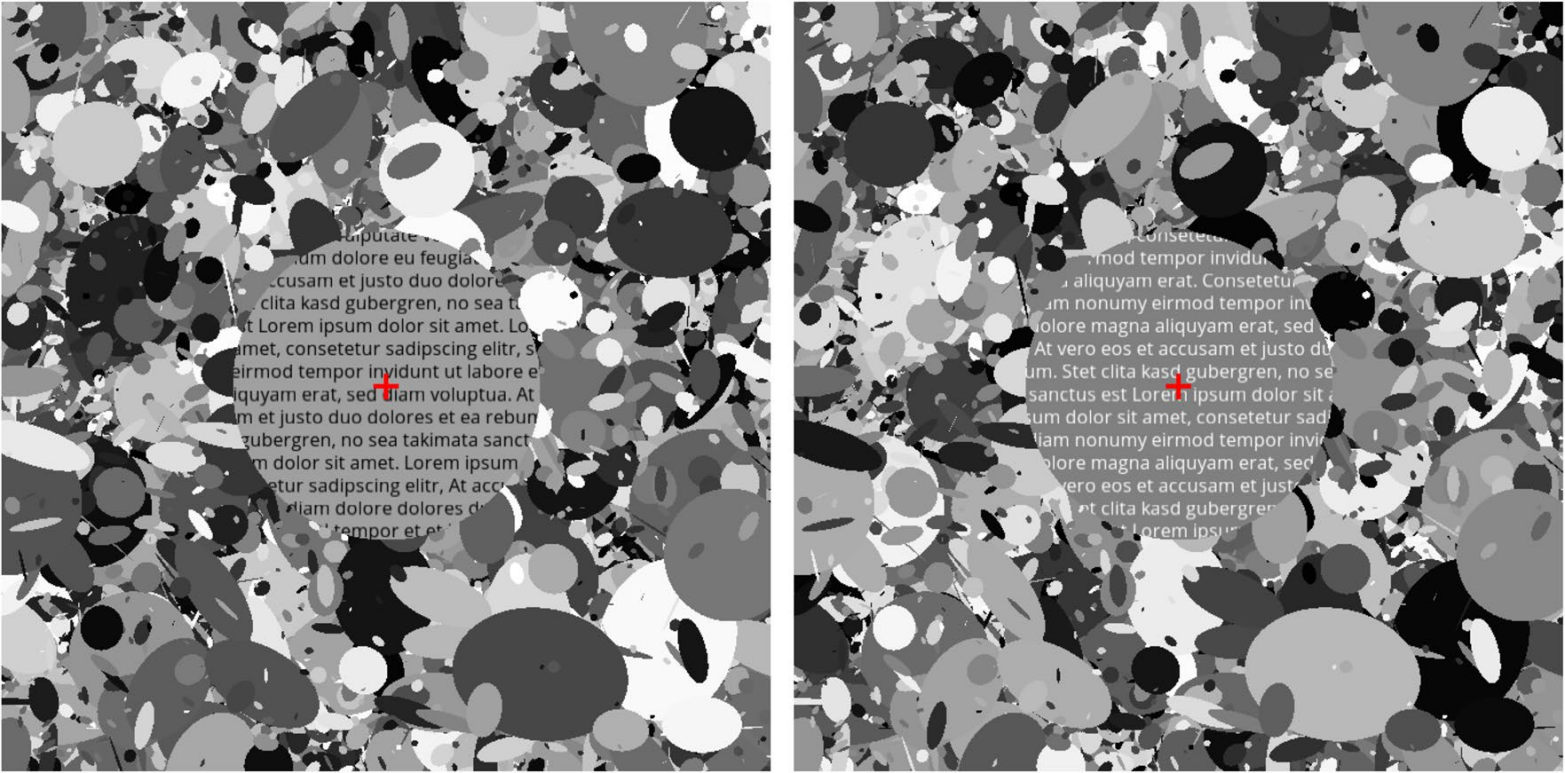
DLS with text component. Example for circle component of dark on bright (left) and bright on dark (right) text elements presented on a dead leaves stimulus [not to scale].

A DLS was created in Python (Python Software Foundation, Beaverton, OR, USA), based on an open-source script^85^ and according to the approach by Panorgias et al.^52^. The stimulus of 23 deg visual angle (670 × 670 pixel) comprised 2000 ellipses of different size that were randomly drawn from a uniform distribution with radii between 2 to 82 pixels. The grayscale value of each ellipse was randomly chosen between 0 (black) and 255 (maximum white). Four different DLS images of both contrast polarities were thus created, matched in luminance, and presented with a contrast reversal of 2 Hz during the recording of pERGs. Text of both contrast polarities was incorporated in this stimulus. To test whether sensitivity to contrast polarity depends on eccentricity, the DLS was overlaid by components of different shape (ring or circle) and size (6 deg or 12 deg), being either filled with text (letter height 0.57 deg, line spacing 0.9 deg, font style Open Sans) of standard or inverted contrast (Fig. 4) or with uniform gray (blank). The text within these areas changed continuously after 16 frames (0.188 s) to simulate the reading procedure. Each stimulus contained a central red fixation cross. Luminance was matched for all conditions, with an average of about 35 cd/m^2^ and room illuminance was kept at about 170 lx.

**Figure 5:**
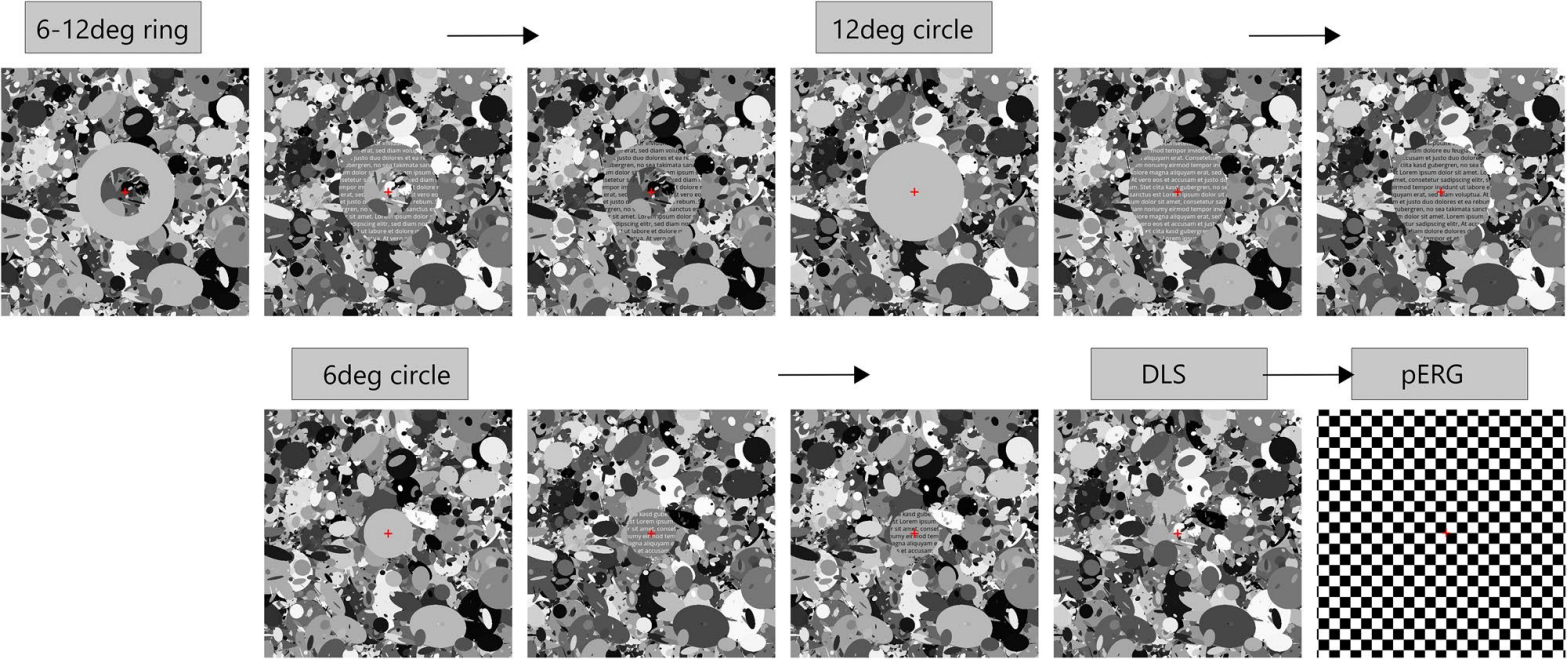
Order of presented stimuli. Stimuli with text components (6–12 deg ring, 12 deg circle, 6 deg circle) were first shown in the blank condition of uniform gray, then with inverted contrast, lastly standard contrast text [not to scale]. All stimuli except for the checkerboard were surrounded by uniform gray (not shown).

The order of stimulus presentation (Fig. 5) was 6–12 deg ring, 12 deg circle, 6 deg circle, each of them in the conditions blank, inverted contrast text, standard contrast text. Then, a clear DLS was shown, lastly followed by the recording of a conventional pERG with a checkerboard stimulus as a reference (2 deg-checkerboard, size 30 × 30 deg visual angle). All stimuli except for the checkerboard were surrounded by uniform gray, covering the remaining display.

### Participants

The study was performed according to the Declaration of Helsinki and approved by the Institutional Review Board of the University of Tuebingen. Volunteers were recruited from the staff of the Institute for Ophthalmic Research Tuebingen. Study procedures and possible risks were explained, and measurements were only performed after having received the volunteer’s informed consent. Monocular and binocular best corrected visual acuity measurements were taken, and based on the spherical equivalent refractive error, the subjects were assigned to the emmetropic (|SER|≤ + 0.5 D) or myopic (SER < −0.5 D) study group.

### Measurement procedure

All ERG recordings were taken under light-adapted, binocular viewing conditions on the right eyes only. Myopic subjects were corrected for far vision using their regular correction method, either spectacle lenses or single vision soft contact lenses. A sterile disposable DTL (Dawson, Trick & Litzkow) electrode^86^ was inserted below the corneal limbus. After skin preparation, the counter electrode was attached to the ipsilateral temple, the ground electrode to the forehead, and both connected to a CE certified electrophysiology system (Espion E2, Diagnosys LLC, Cambridge, UK). For performing the transient pERGs, subjects were seated in front of a CRT monitor (Diamond Plus 230^SB^, Mitsubishi) at 40 cm viewing distance (85 Hz refresh rate; duration 16 frames; 2.66 Hz). Pupils were not dilated, nor was any other topical medication used. For each of the 11 test conditions, 128 sweeps of 188 ms each were recorded, resulting in a total recording duration of about 10 min including short breaks between conditions. Subjects were asked to fixate the central red cross on the display while keeping their head and upright sitting position stable throughout all recordings.

### Data analysis

**Figure 6:**
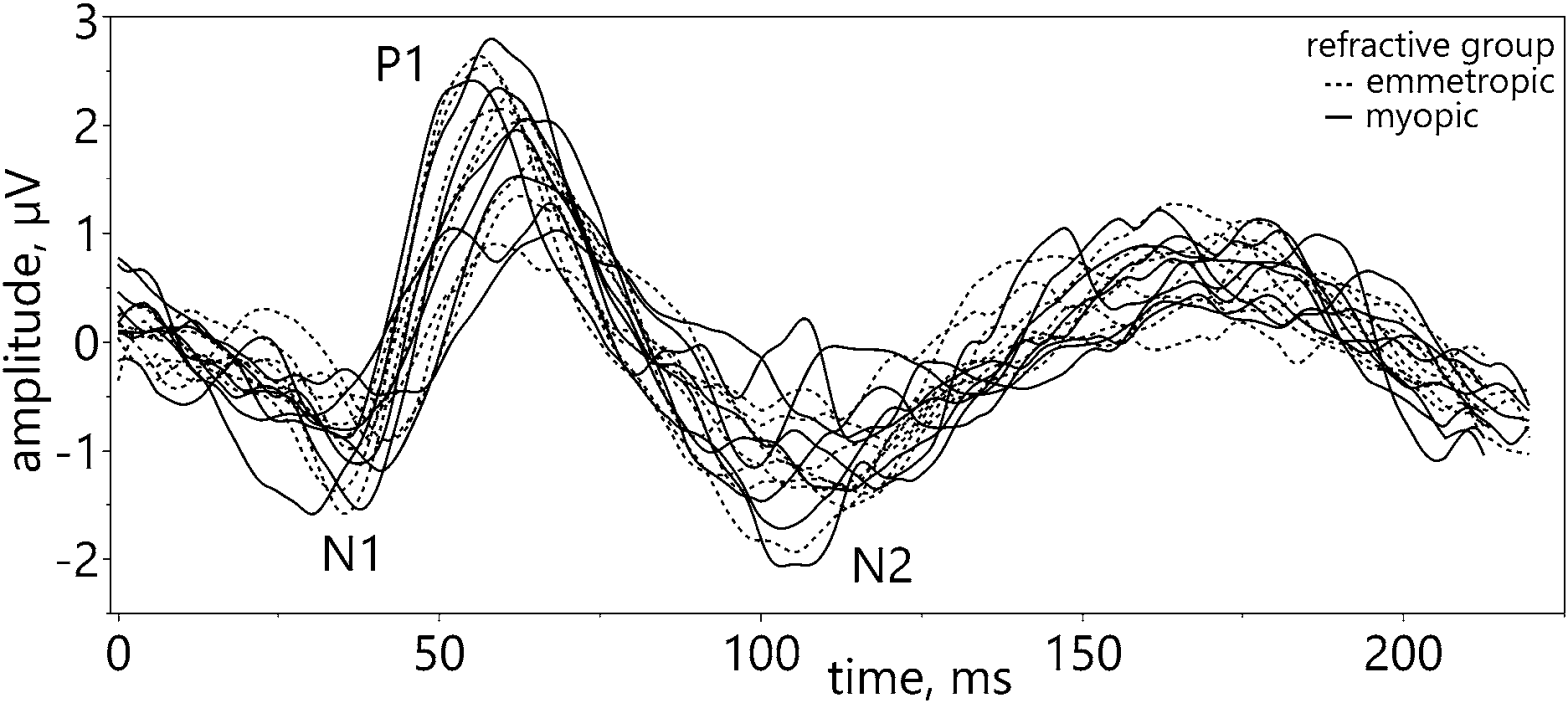
Adjusted pERG waveform nomenclature. Definition of peaks and troughs shown on individual ERG traces recorded with clear DLS. N1: trough at ~ 35 ms; P1: peak at ~ 50 ms; N2: trough at ~ 100 ms.

ERG recordings were bandpass filtered by the amplifier (0.625 to 100 Hz). For analysis, the individual traces were first manually filtered for blinks. Using JMP 16 (SAS Institute GmbH, Heidelberg, Germany), a 3^rd^ order polynomial detrend for normalizing the data^87^ was applied for each sweep, followed by a moving average filter (window ± 12 ms) to reduce the 50 Hz electrical noise. Due to the deviation from the standard protocol^51^ (DLS: broad range of spatial frequencies; contrast between 0–100%; overlaid by masks with text of different contrast polarities or gray; pERG: checkerboard with a single spatial frequency; contrast of 100%), conventional amplitude definitions of the pERG components were renamed, with the first negative component around 35 ms as N1, first positive component around 50 ms as P1, second negative component around 100 ms as N2 (Fig. 6). For the same reason, instead of analyzing amplitudes and latencies, pointwise t-tests^88,89^ were applied to assess the entire response curve changes over time: Groups and conditions were compared using the 95% confidence interval of the mean differences (for comparison of contrast polarity conditions blank vs. standard vs. inverted) or the differences of the means (in case of comparison of refractive groups myopic vs. emmetropic), respectively.

## Supplementary Information

Supplementary Information.

## Data Availability

The datasets generated during and/or analyzed during the current study are available from the corresponding author on reasonable request.

## References

[1] Ip JM (2008). Role of near work in myopia: Findings in a sample of Australian school children. Investig. Ophthalmol. Vis. Sci..

[2] Hughes RPJ, Read SA, Collins MJ, Vincent SJ (2022). Axial elongation during short-term accommodation in myopic and nonmyopic children. Investig. Ophthalmol. Vis. Sci..

[3] Saw S-M (2002). Nearwork in early-onset myopia. Investig. Ophthalmol. Vis. Sci..

[4] Zylbermann R, Landau D, Berson D (1993). The influence of study habits on myopia in Jewish teenagers. J. Pediatr. Ophthalmol. Strabismus.

[5] Huang HM, Chang DST, Wu PC (2015). The association between near work activities and myopia in children—A systematic review and meta-analysis. PLoS ONE.

[6] Kepler, J. *Dioptrik oder Schilderung der Folgen, die sich aus der unlängst gemachten Erfindung der Fernrohre für das Sehen und die sichtbaren Gegenstände ergeben. (original work published in 1611). Trans, from Latin into German by F. Plehn.* (Verlag von Wilhelm Engelmann, 1904).

[7] Rose KA (2008). Myopia, lifestyle, and schooling in students of Chinese ethnicity in Singapore and Sydney. Arch. Ophthalmol..

[8] Verhoeven VJM (2013). Education influences the role of genetics in myopia. Eur. J. Epidemiol..

[9] Morgan IG, Rose KA (2013). Myopia and international educational performance. Ophthalmic Physiol. Opt..

[10] Gwiazda J, Thorn F, Bauer J, Held R (1993). Myopic children show insufficient accommodative response to blur. Investig. Ophthalmol. Vis. Sci..

[11] Vera-Diaz FA, Gwiazda J, Thorn F, Held R (2004). Increased accommodation following adaptation to image blur in myopes. J. Vis..

[12] Day M, Strang NC, Seidel D, Gray LS, Mallen EAH (2006). Refractive group differences in accommodation microfluctuations with changing accommodation stimulus. Ophthalmic Physiol. Opt..

[13] Harb E, Thorn F, Troilo D (2006). Characteristics of accommodative behavior during sustained reading in emmetropes and myopes. Vision Res..

[14] McBrien NA, Millodot M (1987). The relationship between tonic accommodation and refractive error. Investig. Ophthalmol. Vis. Sci..

[15] Buckhurst H, Gilmartin B, Cubbidge RP, Nagra M, Logan NS (2013). Ocular biometric correlates of ciliary muscle thickness in human myopia. Ophthalmic Physiol. Opt..

[16] Wagner S, Zrenner E, Strasser T (2019). Emmetropes and myopes differ little in their accommodation dynamics but strongly in their ciliary muscle morphology. Vision Res..

[17] Bailey MD, Sinnott LT, Mutti DO (2008). Ciliary body thickness and refractive error in children. Investig. Ophthalmol. Vis. Sci..

[18] Jeon S, Lee WK, Lee K, Moon NJ (2012). Diminished ciliary muscle movement on accommodation in myopia. Exp. Eye Res..

[19] Mutti DO (2010). Hereditary and environmental contributions to emmetropization and myopia. Optom. Vis. Sci..

[20] Flitcroft DI, Harb EN, Wildsoet CF (2020). The spatial frequency content of urban and indoor environments as a potential risk factor for myopia development. Investig. Ophthalmol. Vis. Sci..

[21] Flitcroft DI (2012). The complex interactions of retinal, optical and environmental factors in myopia aetiology. Prog. Retin. Eye Res..

[22] Choi KY, Mok AYT, Do CW, Lee PH, Chan HHL (2020). The diversified defocus profile of the near-work environment and myopia development. Ophthalmic Physiol. Opt..

[23] Choi KY, Chan SSH, Chan HHL (2021). The effect of spatially-related environmental risk factors in visual scenes on myopia. Clin. Exp. Optom..

[24] Aleman AC, Wang M, Schaeffel F (2018). Reading and myopia: contrast polarity matters. Sci. Rep..

[25] Schiller PH, Sandell JH, Maunsell JHR (1995). The ON and OFF channels of the mammalian visual system. Prog. Retin. Eye Res..

[26] Werblin FS, Dowling JE (1969). Organization of the retina of the mudpuppy, Necturus maculosus. II. Intracellular recording. J. Neurophysiol..

[27] Kuffler SW (1953). Discharge patterns and functional organization of mammalian retina. J. Neurophysiol..

[28] Dacey DM, Petersen MR (1992). Dendritic field size and morphology of midget and parasol ganglion cells of the human retina. Proc. Natl. Acad. Sci. USA.

[29] Zemon V, Gordon J, Welch J (1988). Asymmetries in ON and OFF visual pathways of humans revealed using contrast-evoked cortical potentials. Vis. Neurosci..

[30] Chichilnisky EJ, Kalmar RS (2002). Functional asymmetries in ON and OFF ganglion cells of primate retina. J. Neurosci..

[31] Pardue MT (2008). High susceptibility to experimental myopia in a mouse model with a retinal on pathway defect. Investig. Ophthalmol. Vis. Sci..

[32] Crewther DP, Crewther SG (2002). Refractive compensation to optical defocus depends on the temporal profile of luminance modulation of the environment. NeuroReport.

[33] Crewther SG, Crewther DP (2003). Inhibition of retinal ON/OFF systems differentially affects refractive compensation to defocus. NeuroReport.

[34] Chakraborty R (2015). ON pathway mutations increase susceptibility to form-deprivation myopia. Exp. Eye Res..

[35] Wang M, Aleman AC, Schaeffel F (2019). Probing the potency of artificial dynamic ON or OFF stimuli to inhibit myopia development. Investig. Ophthalmol. Vis. Sci..

[36] Troilo D, Nickla DL, Wildsoet CF (2000). Choroidal thickness changes during altered eye growth and refractive state in a primate. Investig. Ophthalmol. Vis. Sci..

[37] Zhang S (2019). Changes in choroidal thickness and choroidal blood perfusion in guinea pig myopia. Investig. Ophthalmol. Vis. Sci..

[38] Lan W, Feldkaemper M, Schaeffel F (2013). Bright light induces choroidal thickening in chickens. Optom. Vis. Sci..

[39] Wallman J (1995). Moving the retina: choroidal modulation of refractive state. Vision Res..

[40] Wang D (2016). Optical defocus rapidly changes choroidal thickness in schoolchildren. PLoS ONE.

[41] Hoseini-Yazdi H, Read SA, Alonso-Caneiro D, Collins MJ (2021). Retinal OFF-pathway overstimulation leads to greater accommodation-induced choroidal thinning. Investig. Ophthalmol. Vis. Sci..

[42] Hogue W, Taylor CP (2021). Axial length is associated with individual differences in ON- and OFF-pattern detection. Invest. Ophthalmol. Vis. Sci..

[43] Oner A, Gumus K, Arda H, Karakucuk S, Mirza E (2009). Pattern electroretinographic recordings in eyes with myopia. Eye Contact Lens.

[44] Ho WC, Kee CS, Chan HHL (2012). Myopia progression in children is linked with reduced foveal mfERG response. Investig. Ophthalmol. Vis. Sci..

[45] Chen JC, Brown B, Schmid KL (2006). Delayed mfERG responses in myopia. Vision Res..

[46] Li SZ-C (2017). Subclinical decrease in central inner retinal activity is associated with myopia development in children. Investig. Ophthalmol. Vis. Sci..

[47] Kong AW, Santina Della L, Ou Y (2020). Probing ON and OFF retinal pathways in glaucoma using electroretinography. Transl. Vis. Sci. Technol..

[48] Pangeni G, Lämmer R, Tornow RP, Horn FK, Kremers J (2012). On- and off-response ERGs elicited by sawtooth stimuli in normal subjects and glaucoma patients. Doc. Ophthalmol..

[49] Norcia AM, Yakovleva A, Hung B, Goldberg JL (2020). Dynamics of contrast decrement and increment responses in human visual cortex. Transl. Vis. Sci. Technol..

[50] Goldberg JL, Yakovleva A, Hung B, Norcia A (2018). Dynamics of human ON and OFF visual pathways. Invest. Ophthalmol. Vis. Sci..

[51] Bach M (2013). ISCEV standard for clinical pattern electroretinography (PERG): 2012 update. Doc. Ophthalmol..

[52] Panorgias A (2021). Retinal responses to simulated optical blur using a novel dead leaves ERG stimulus. Investig. Ophthalmol. Vis. Sci..

[53] Ho WC (2012). Sign-dependent changes in retinal electrical activity with positive and negative defocus in the human eye. Vision Res..

[54] Wallman J, Winawer J (2004). Homeostasis of eye growth and the question of myopia. Neuron.

[55] Winawer J, Zhu X, Choi J, Wallman J (2005). Ocular compensation for alternating myopic and hyperopic defocus. Vision Res..

[56] Rosenfield M, Abraham-Cohen JA (1999). Blur sensitivity in myopes. Optom. Vis. Sci..

[57] Maiello G, Walker L, Bex PJ, Vera-Diaz FA (2017). Blur perception throughout the visual field in myopia and emmetropia. J. Vis..

[58] Schmid KL, Iskander RD, Li RWH, Edwards MH, Lew JKF (2002). Blur detection thresholds in childhood myopia: Single and dual target presentation. Vision Res..

[59] Xu Z (2022). Assessing the contrast sensitivity function in myopic parafovea: A quick contrast sensitivity functions study. Front. Neurosci..

[60] Liu Y, Wildsoet C (2011). The effect of two-zone concentric bifocal spectacle lenses on refractive error development and eye growth in young chicks. Investig. Opthalmology Vis. Sci..

[61] Smith EL, Hung L-F, Huang J (2009). Relative peripheral hyperopic defocus alters central refractive development in infant monkeys. Vision Res..

[62] Stoimenova BD, Kurtev A, Georgiev M (1995). Contrast sensitivity in emmetropes and myopes using on- and off-stimulation. Vision Res..

[63] Stoimenova BD (2007). The effect of myopia on contrast thresholds. Investig. Ophthalmol. Vis. Sci..

[64] Ratliff CP, Borghuis BG, Kao YH, Sterling P, Balasubramanian V (2010). Retina is structured to process an excess of darkness in natural scenes. Proc. Natl. Acad. Sci. USA.

[65] Thorn F, Corwin TR, Comerford JP (1986). High myopia does not affect contrast sensitivity. Curr. Eye Res..

[66] Tedja MS (2018). Genome-wide association meta-analysis highlights light-induced signaling as a driver for refractive error. Nat. Genet..

[67] Verhoeven VJM (2013). Genome-wide meta-analyses of multi-ethnic cohorts identify multiple new susceptibility loci for refractive error and myopia. Nat. Genet..

[68] Hysi PG (2020). Meta-analysis of 542,934 subjects of European ancestry identifies new genes and mechanisms predisposing to refractive error and myopia. Nat. Genet..

[69] Tedja MS (2019). IMI—Myopia genetics report. Investig. Ophthalmol. Vis. Sci..

[70] Clark R (2022). Education interacts with genetic variants near GJD2, RBFOX1, LAMA2, KCNQ5 and LRRC4C to confer susceptibility to myopia. PLoS Genet..

[71] Aulhorn E (1953). Über Fixationsbreite und Fixationsfrequenz beim Lesen gerichteter Konturen. Pflugers Arch. Gesamte Physiol. Menschen Tiere.

[72] Trauzettel-Klosinski S (2002). Reading disorders due to visual field defects: A neuro-ophthalmological view. Neuro-Ophthalmology.

[73] van Alphen GWHM (1986). Choroidal stress and emmetropization. Vision Res..

[74] Wagner S, Schaeffel F, Zrenner E, Straßer T (2019). Prolonged nearwork affects the ciliary muscle morphology. Exp. Eye Res..

[75] Wagner S, Strasser T (2022). Does reading text with inverted contrast affect the ciliary muscle structure of emmetropic and myopic eyes?. Investig. Ophthalmol. Vis. Sci..

[76] Lee AB, Mumford D, Huang J (2001). Occlusion models for natural images: A statistical study of a scale-invariant dead leaves model. Int. J. Comput. Vis..

[77] Wienbar S, Schwartz GW (2018). The dynamic receptive fields of retinal ganglion cells. Prog. Retin. Eye Res..

[78] Watson AB (2014). A formula for human retinal ganglion cell receptive field density as a function of visual field location. J. Vis..

[79] Miura G, Wang MH, Ivers KM, Frishman LJ (2009). Retinal pathway origins of the pattern ERG of the mouse. Exp. Eye Res..

[80] Luo X, Frishman LJ (2011). Retinal pathway origins of the pattern electroretinogram (PERG). Investig. Ophthalmol. Vis. Sci..

[81] Slaughter MM, Miller RF (1981). 2-amino-4-phosphonobutyric acid: A new pharmacological tool for retina research. Science (80-).

[82] Khan NW (2005). Primate retinal signaling pathways: Suppressing on-pathway activity in monkey with glutamate analogues mimics human CSNB1-NYX genetic night blindness. J. Neurophysiol..

[83] Miyake Y, Yagasaki K, Horiguchi M, Kawase Y (1987). On- and off-responses in photopic electroretinogram in complete and incomplete types of congenital stationary night blindness. Jpn. J. Ophthalmol..

[84] Chaudron S, Di Gioia R, Gemo M (2018). Young children (0–8) and digital technology: a qualitative study across Europe. JRC Sci. Policy Rep..

[85] Peyre, G. Toolbox image. at https://de.mathworks.com/matlabcentral/fileexchange/16201-toolbox-image (2022).

[86] Dawson WW, Trick GLT, Litzkow CA (1979). Improved electrode for electroretinography. Investig. Ophthalmol. Vis. Sci..

[87] Tang J, Hui F, Coote M, Crowston JG, Hadoux X (2018). Baseline detrending for the photopic negative response. Transl. Vis. Sci. Technol..

[88] Zhang C, Peng H, Zhang JT (2010). Two samples tests for functional data. Commun. Stat. - Theory Methods.

[89] Shorter KA, Polk JD, Rosengren KS, Hsiao-Wecksler ET (2008). A new approach to detecting asymmetries in gait. Clin. Biomech..

