# Supplementary material for: Impact of text contrast polarity on the retinal activity in myopes and emmetropes using modified pattern ERG
Source: Sci Rep. 2023 Jul 9;13:11101. doi: 10.1038/s41598-023-38192-9 (PMC10330186; doi:10.1038/s41598-023-38192-9)
Supplement: Supplementary file 1 — Supplementary Information. [file 41598_2023_38192_MOESM1_ESM.pdf]

Supplementary Information

Impact of text contrast polarity on the retinal activity in myopes and emmetropes using modified pattern ERG.

Sandra Wagner, Torsten Strasser

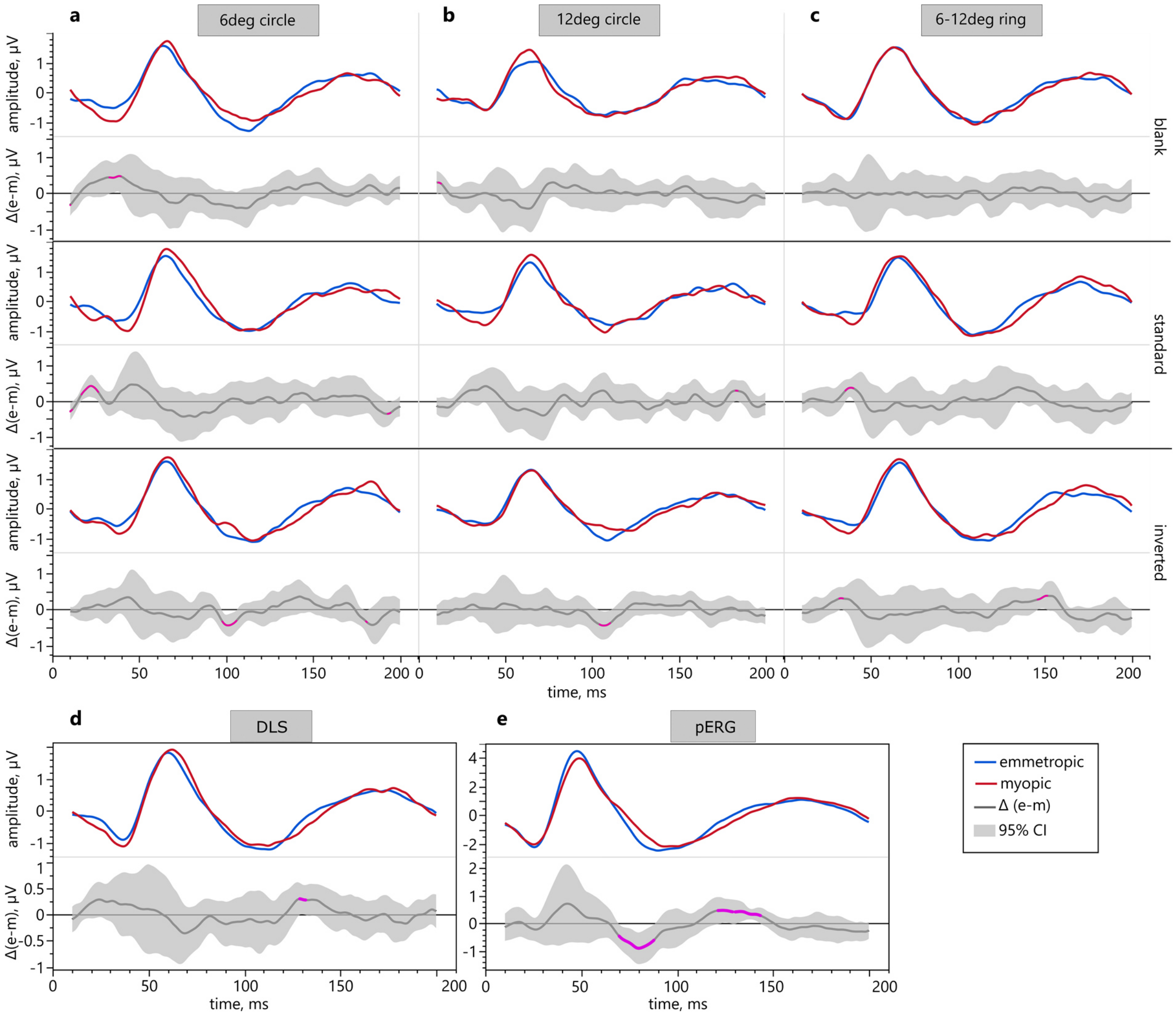

Supplementary Figure 1: Statistical analysis of comparison between refractive groups. Statistical analysis of differences between emmetropic and myopic subjects for different retinal eccentricities and contrast polarities (a-c), as well as for DLS clear (d) and standard pERG responses (e). Upper plots depict the retinal responses for the two study groups, bottom plots show difference between these curves, respectively. Purple highlights mark substantial differences using the 95 % confidence intervals.

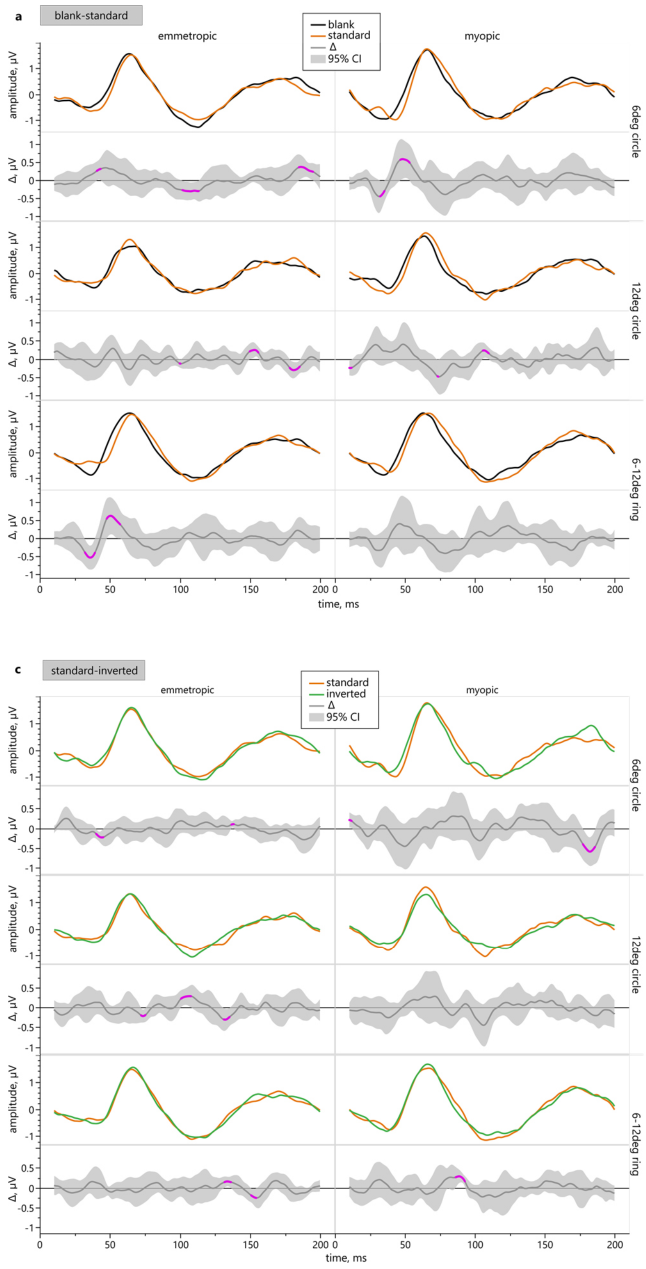

Supplementary Figure 2: Statistical analysis of comparison between contrast polarities. Statistical analysis of differences between contrast polarities in the three tested masks. a: Difference between blank and standard contrast condition; b: Difference between blank and inverted contrast condition; c: Difference between standard and inverted contrast condition. Upper plots depict the retinal responses for the two conditions, bottom plots show difference between these curves, respectively. Purple highlights mark substantial differences using the 95 % confidence intervals.
